# Trehalose 6-phosphate synthase gene *rdtps1* contributes to thermal acclimation in *Rhyzopertha dominica*

**DOI:** 10.1186/s12864-024-10028-4

**Published:** 2024-02-13

**Authors:** Dingrong Xue, Yan Yang, Liwei Fang, Shibo Wang, Yi Wu

**Affiliations:** 1 National Engineering Research Center of Grain Storage and Logistics, Academy of National Food and Strategic Reserves Administration, No. 11 Baiwanzhuang Street, Xicheng District, 100037 Beijing, China; 2 Henan Collaborative Innovation Center for Grain Storage Security, School of Food and Strategic Reserves, Henan University of Technology, 450001 Zhengzhou, China; 3 Department of Microbiology and Immunology, University of Illinois Chicago, 60612 Chicago, USA; 4 School of Health Science and Engineering, University of Shanghai for Science and Technology, 200093 Shanghai, China

**Keywords:** *Rhyzopertha dominica*, Temperature, Transcriptome analysis, Trehalose 6-phosphate synthase, RNAi

## Abstract

**Background:**

The lesser grain borer (*Rhyzopertha dominica*), a worldwide primary pest of stored grain, causes serious economic losses and threatens stored food safety. *R. dominica* can respond to changes in temperature, especially the adaptability to heat. In this study, transcriptome analysis of *R. dominica* exposed to different temperatures was performed to elucidate differences in gene expression and the underling molecular mechanism.

**Results:**

Isoform-sequencing generated 17,721,200 raw reads and yielded 20,416 full-length transcripts. A total of 18,880 (92.48%) transcripts were annotated. We extracted RNA from *R. dominica* reared at 5 °C (cold stress), 15 °C (cold stress), 27 °C (ambient temperature) and 40 °C (heat stress) for RNA-seq. Compared to those of control insects reared at 27 °C, 119, 342, and 875 differentially expressed genes (DEGs) were identified at 5 °C, 15 °C, and 40 °C, respectively. Kyoto Encyclopedia of Genes and Genomes (KEGG) analysis revealed that pathways associated with “fatty acid metabolism”, “fatty acid biosynthesis”, “AMPK signaling pathway”, “neuroactive ligand receptor interaction”, and “longevity regulating pathway-multiple species” were significantly enriched. The functional annotation revealed that the genes encoding heat shock proteins (HSPs), fatty acid synthase (FAS), phospholipases (PLA), trehalose transporter (TPST), trehalose 6-phosphate synthase (TPS), and vitellogenin (Vg) were most likely involved in temperature regulation, which was also validated by RT-qPCR. Seven candidate genes (*rdhsp1*, *rdfas1*, *rdpla1*, *rdtpst1*, *rdtps1*, *rdvg1*, and *rdP450*) were silenced in the RNA interference (RNAi) assay. RNAi of each candidate gene suggested that inhibiting *rdtps1* expression significantly decreased the trehalose level and survival rate of *R. dominica* at 40 °C.

**Conclusions:**

These results indicated that trehalose contributes to the high temperature resistance of *R. dominica*. Our study elucidates the molecular mechanisms underlying heat tolerance and provides a potential target for the pest management in *R. dominica*.

**Supplementary Information:**

The online version contains supplementary material available at 10.1186/s12864-024-10028-4.

## Background

The lesser grain borer, *Rhyzopertha dominica* (Fabricius) (Coleoptera: Bostrychidae), is one of the most devastating pests of stored grain. *R. dominica* displays remarkable adaptability, attributed to its resistance to desiccation, heat, and hypoxia [1–4]. Both the adults and larvae of *R. dominica* cause damage inside the grain, resulting in a decrease in grain quality [5–7]. In addition, the frass produced by insects can also promote populations of secondary stored product pests and microorganisms [8]. The grains contaminated by *R. dominica* emit a severe amount of off-odor, rendering them unfit for human consumption [9]. Therefore, *R. dominica* causes serious economic losses and constitutes a grave threat to food safety.

Temperature is one of the most prominent abiotic factors influencing the ecological distribution, life cycle, growth, development, reproduction, phylogenetic evolution, and immune response of organisms [10, 11]. However, insects have various mechanisms to adapt to changes in ambient temperature [12–15]. Heat shock proteins (HSPs) are critical factors in the response to temperature stress. For example, the expression of HSP genes significantly differed under temperature stress in *Glyphodes pyloalis* and *Callosobruchus chinensis* [15, 16]. In *Galeruca daurica*, the genes encoding cuticle protein, P450, clock protein, fatty acid synthase, and fatty acyl-CoA reductase were upregulated under cold stress [17]. In addition, regulating the level of trehalose is one of the mechanisms by which insects counteract temperature changes [18, 19]. Trehalose, a ubiquitous nonreducing disaccharide, acts as a metabolic substance in the stress response of many organisms. Its levels change in response to external conditions such as heat, cold, desiccation and anoxia [20–25]. Many insect species, including *Monochamus alternatus* and *Helicoverpa armigera*, use trehalose as a protective agent to enhance cold resistance [26, 27]. Studies have shown that trehalose regulates the viscosity of the cytoplasm during thermal stimulation to maintain cytoplasmic homeostasis and stabilize the reaction rate in cells [28, 29]. Trehalose is predominantly synthesized via the trehalose-6-phosphate synthase (TPS) and trehalose-6-phosphate phosphatase (TPP) pathways in insects. In this pathway, uridine diphosphate glucose (UDP-glucose) and 6-phosphate glucose can generate 6-phosphate trehalose under the action of TPS, while 6-phosphate trehalose can detach the phosphate group and ultimately produce trehalose under the action of TPP [30]. In addition, trehalose can be synthesized from some insect species by TPS alone. This type of TPS has an N-terminal TPS (glycosyltransferase family 20) domain that catalyzes the production of trehalose-6-phosphate using glucose 6-phosphate and UDP-glucose as substrates, whereas the C-terminal TPS (trehalose-phosphatases) domain then dephosphorylates trehalose-6-phosphate, generating trehalose and orthophosphate [31, 32]. Therefore, TPSs play a vital role in trehalose synthesis and in the regulatory pathway of the temperature response.

The rapid development of sequencing technology has made it possible to analyze differentially expressed genes (DEGs) that may be involved in temperature response mechanisms. Given that the genome sequence was unavailable during our study, full-length isoform sequencing (ISO-Seq) facilitated the profiling of the genome-independent transcriptome. Short-read RNA-Seq using high-throughput next-generation sequencing (NGS) technology enables the analysis of DEGs [33]. RNA interference (RNAi) is a widely used technology in which target genes are silenced through double-stranded DNA (dsDNA). RNAi is commonly employed in gene function research and a potential new method for pest control [34, 35]. In this study, we constructed a full-length transcriptomic database and identified DEGs related to temperature regulation in *R. dominica.* The DEGs were further validated using RT-qPCR and RNAi. Taken together, our findings demonstrated that the accumulation of trehalose contributes to resistance to high temperature stress, and the trehalose 6-phosphate synthase gene *rdtps1* positively regulates trehalose levels during thermal acclimation in *R. dominica*.

## Results

### ***Rhyzopertha dominica*adapted to a wide range of temperature conditions**

**Fig. 1:**
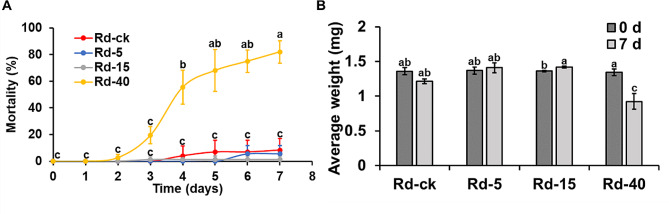
*Rhyzopertha dominica* adapted to a wide range of temperature conditions. **(A)** Mortality of *R. dominica* at different temperatures. **(B)** The average weight of *R. dominica* reared at different temperatures for 7 days. Rd-ck, Rd-5, Rd-15, and Rd-40 indicate *R. dominica* reared at 27 °C, 5 °C, 15 °C, and 40 °C, respectively. Different letters (a to d) above the bars indicate a significant difference (*P* < 0.05) by the Turky test

To determine the mortality rate of *R. dominica* at different temperatures, we reared the insects at a suitable temperature (27 °C), low temperatures (5 and 15 °C) and a high temperature (40 °C) for 7 days. *R. dominica* kept at 27 °C were used as the control group (Rd-ck), whereas *R. dominica* kept at 5 °C, 15 °C, and 40 °C were the treatment groups (Rd-5, Rd-15, and Rd-40). The mortality rates of Rd-ck, Rd-5, and Rd-15 were less than 10% after seven days, while Rd-ck, the group reared at ambient temperature for *R. dominica* growth and development, had the highest mortality rate among these three groups. Although 40 °C is a relatively high temperature for most stored grain pests and the mortality rate of Rd-40 reached 82% on day 7, the mortality of the pests did not exceed 10% until day 3 (Fig. 1A). In addition, we measured the weight of the adults before and after temperature treatment (Fig. 1B). The average weight of Rd-40 decreased after 7 days, while that of the Rd-ck and Rd-5 did not significantly change. Since the mortality rate at different temperatures did not change significantly during the first 48 h, the time point was used for transcriptome sequencing.

### Full-length transcriptome sequencing provides a reference for differentially expressed genes analysis

**Fig. 2:**
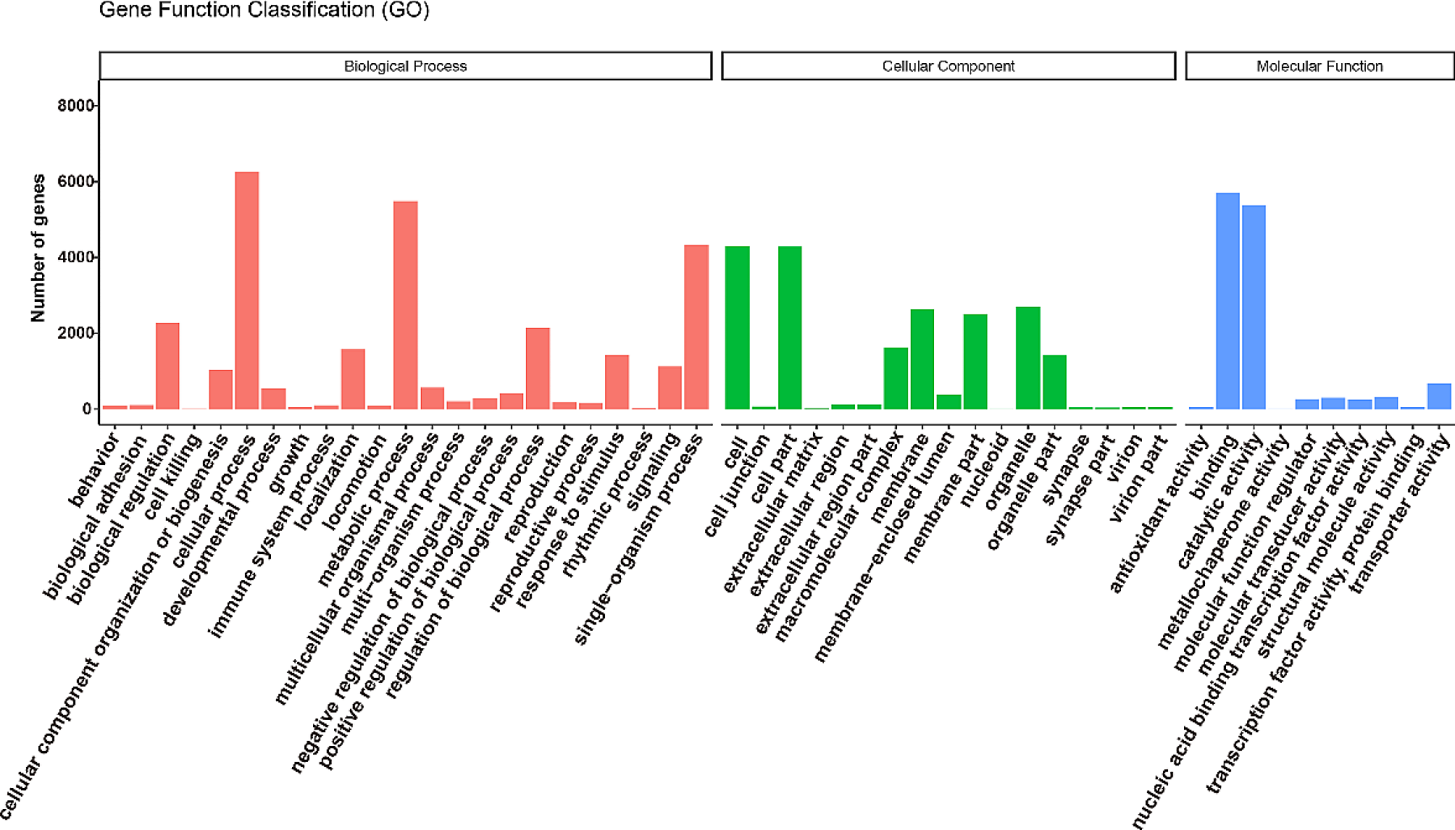
GO classification comprised biological process, molecular function and cellular component of all identified isoforms from the reference transcriptome of *Rhyzopertha dominica*

Since there was no available genome sequence during this study, the full-length transcriptome was sequenced to obtain full-length transcripts as reference sequences. Isoform sequencing generated 17,721,200 raw reads. After clustering, polishing, and removing redundant isoforms, we obtained 20,416 full-length transcripts. To characterize the functional information of the full-length transcripts, we aligned the transcript sequences with public databases, including Swiss-Prot, NCBI nucleotide sequences (NT), NCBI non-redundant protein sequences (NR), Clusters of orthologous groups for Eukaryote (KOG), Kyoto Encyclopedia of Genes and Genomes (KEGG) KEGG, and Gene Ontology (GO). In total, 18,880 (92.48%) of the non-redundant high-quality transcripts (also known as unigenes) could be functionally annotated using Swiss-Prot (16,075), NT (6,795), NR (18,746), KOG (14,663), KEGG (13,201), and GO (11,795) (Fig. S1A). The four databases with the most annotations that provided 18,824 transcript annotations were NR, Swiss-Prot, KOG, and KEGG. Some of the transcripts were mapped to more than one database, and 11,510 transcripts were simultaneously mapped to the NR, Swiss-Prot, KOG, and KEGG databeases (Fig. S1B). According to the KEGG Orthology database (KO) of KEGG, 2,135 (14.88%), 2,137 (14.90), 2,460 (17.15%), 3,687 (25.70%), and 3,927 (27.37%) of the isoforms were associated with cellular processes (A), environmental information processing (B), genetic information processing (C), metabolism (D), and organismal systems (E), respectively (Fig. S1C). Additionally, GO annotation incorporates three categories, namely, biological process (19,619 isoforms, 45.32%), cellular component (14,338 isoforms, 33.12%), and molecular function (9,330 isoforms, 21.56%) (Fig. 2). To further profile the functional annotations of all the isoforms, we annotated 14,663 transcripts with 16,550 KOG functions in 4 categories and 25 subcategories (Fig. S1D). The four categories were “cellular processes and signaling” (6,468 isoforms, 39.08%), “information storage and processing” (3,256 isoforms, 19.67%), “metabolism” (3,465 isoforms, 20.94%), and “poorly characterized” (3,361 isoforms, 20.31%).

### Identification and functional profiles of differentially expressed genes under temperature stress

**Fig. 3:**
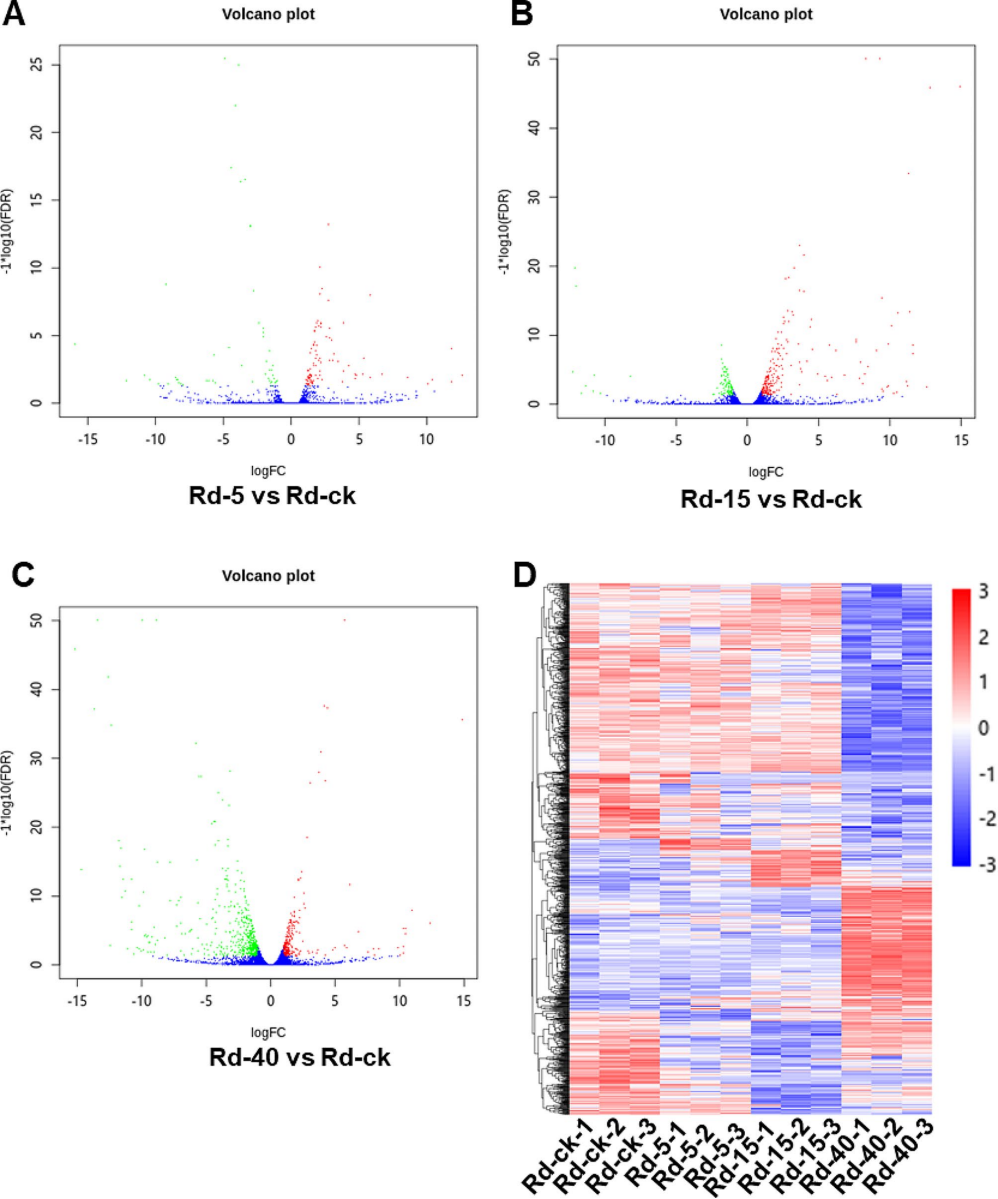
Differentially expressed genes (DEGs) of *Rhyzopertha dominica* exposed to different temperatures. **(A-C)** DEGs of Rd-5, Rd-15, and Rd-40 compared to Rd-ck. The x-axis represents the log_2_ (fold-change) values under the mean normalized expression of all isoforms (y-axis). Red dots indicate the upregulated DEGs, and green dots represent the downregulated DEGs. **(D)** Heatmap of DEGs in Rd-5, Rd-15, and Rd-40 compared to Rd-ck. Rd-ck, Rd-5, Rd-15 and Rd-40 indicate *R. dominica* reared at 27 °C, 5 °C, 15 °C, and 40 °C, respectively

To characterize the functional genes of *R. dominica* in response to temperature changes, Illumina libraries of Rd-ck, Rd-5, Rd-15, and Rd-40 were generated (Table 1). Compared to Rd-ck, there were 119, 342, and 875 DEGs identified in Rd-5, Rd-15, and Rd-40, respectively. Thirty-six genes were up-regulated and 83 genes were down-regulated at 5 °C (Fig. 3A). 107 genes were up-regulated and 235 genes were down-regulated at 15 °C (Fig. 3B). 264 genes were up-regulated and 611 genes were down-regulated at 40 °C (Fig. 3C). A total of 1142 DEGs were identified and used for heatmap analysis (Fig. 3D). The results demonstrated significant intergroup differences, particularly with respect to the different expression patterns observed for Rd-40.

**Table 1:** Information of RNA-Seq clean reads mapped with the ISO-Seq transcriptome

Sample	Total number of reads	Mapped reads (ratio)	Uniquely mapped reads (ratio)	Multi mapped reads (ratio)
Rd-ck-1	27,219,928	22,753,138 (83.59%)	7,959,107 (29.24%)	14,794,031 (54.35%)
Rd-ck-2	23,664,642	20,067,616 (84.80%)	7,037,865 (29.74%)	13,029,752 (55.06%)
Rd-ck-3	38,676,635	32,716,566 (84.59%)	11,552,711(29.87%)	21,163,855 (54.72%)
Rd-5-1	37,023,641	30,814,776 (83.23%)	11,321,829 (30.58%)	19,492,947 (52.64%)
Rd-5-2	43,705,288	36,699,330 (83.97%)	13,037,287 (29.83%)	23,662,043 (54.15%)
Rd-5-3	35,002,416	29,671,548 (84.77%)	10,605,732 (30.30%)	19,065,816 (54.47%)
Rd-15-1	47,676,396	39,976,658 (83.85%)	14,245,707 (29.88%)	25,730,951 (53.97%)
Rd-15-2	24,974,596	21,228,407 (85.00%)	7,574,795 (30.33%)	13,653,612 (54.67%)
Rd-15-3	42,353,666	35,564,373 (83.97%)	12,523,979 (29.57%)	23,040,394 (54.40%)
Rd-40-1	28,489,413	23,663,306 (83.06%)	8,791,833 (30.86%)	14,871,474 (52.20%)
Rd-40-2	20,168,430	16,739,797 (83.00%)	6,240,112 (30.94%)	10,499,685 (52.06%)
Rd-40-3	37,023,641	30,814,776 (83.23%)	11,321,829 (30.58%)	19,492,947 (52.64%)

Notes: Rd-ck (*R. dominica* reared at 27 °C), Rd-5 (*R. dominica* reared at 5 °C), Rd-15 (*R. dominica* reared at 15 °C), and Rd-40 (*R. dominica* reared at 40 °C)

**Fig. 4:**
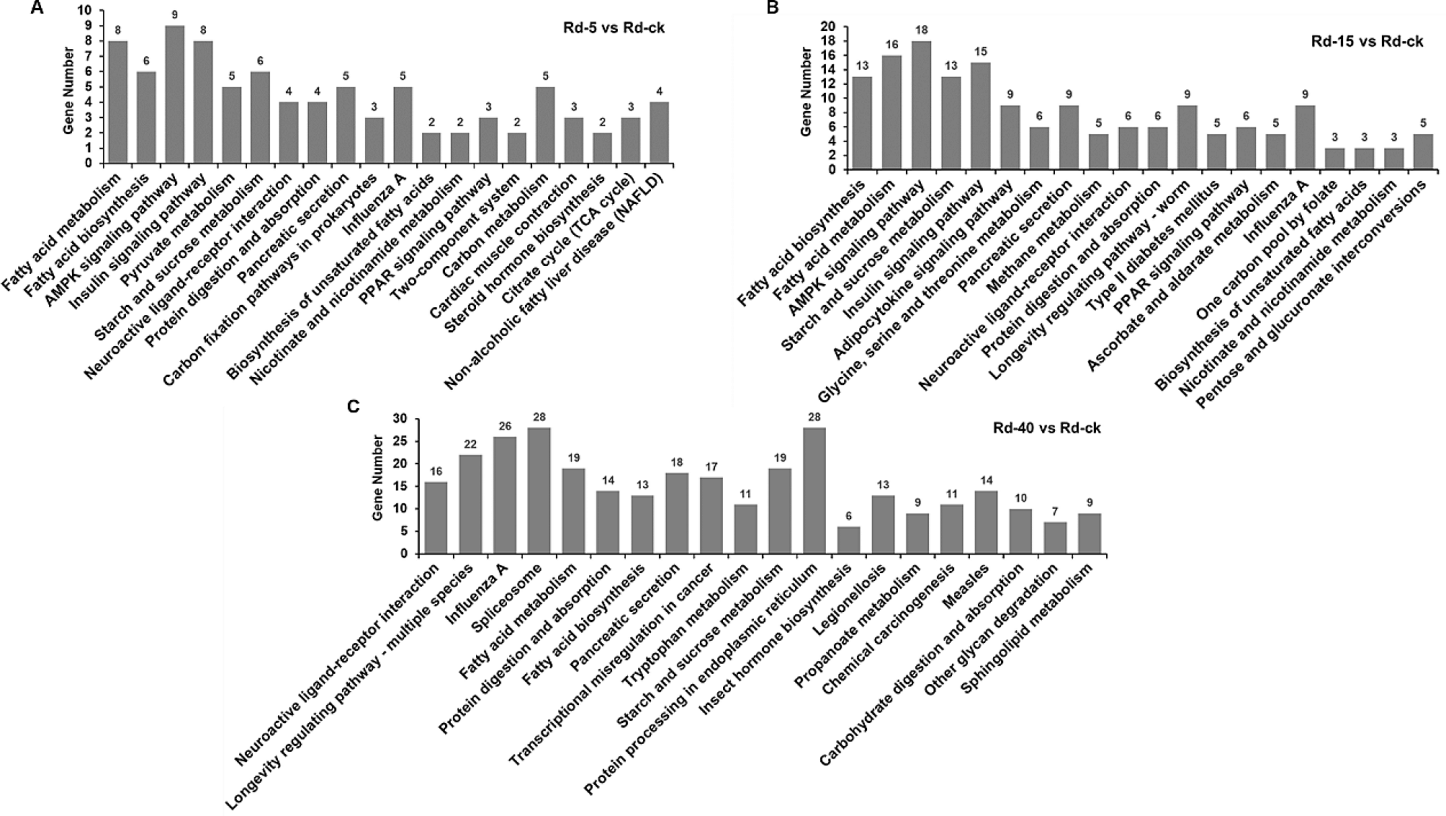
The top 20 significantly enriched KEGG (Kyoto Encyclopedia of Genes and Genomes) pathways among Differentially expressed genes (DEGs) in **A** (5 °C vs. 27 °C), **B** (15 °C vs. 27 °C) and **C** (40 °C vs. 27 °C). The X axis indicates the top 20 terms enriched in KEGG, and the Y axis represents the number of DEGs annotated under that term

Metabolic pathways were systematically analyzed to construct a network of gene expression information via KEGG analysis [36]. The top 20 most reliable enrichment pathways are shown in Fig. 4. Under cold stress, 124 and 179 pathways were enriched in Rd-5 and Rd-15, respectively. The primary components were “fatty acid metabolism”, “fatty acid biosynthesis”, “AMPK signaling pathway”, “insulin signaling pathway”, and “starch and sucrose metabolism” (Fig. 4A-B). Under heat stress, “neuroactive ligand-receptor interaction”, “longevity regulating pathway-multiple species”, “influenza A”, “spliceosome”, and “fatty acid metabolism” were the primary components (Fig. 4C). Thirty-four genes were coregulated among Rd-5, Rd-15, and Rd-40, which accounted for 3% of the total DEGs (Fig. S2). These genes included mainly the genes encoding fatty acid synthase (FAS) and vitellogenin (Vg), and their expression levels were inhibited under temperature stress. Genes with high expression under 40 °C conditions were primarily associated with heat shock proteins (HSPs), ATP-binding cassettes, and phospholipases, while genes with decreased expression were mainly related to serine protease activity regulation. A total of 117 genes (7.6%) were coregulated between Rd-15 and Rd-40, including genes related to the trehalose pathway, vitellogenin, and cytochrome P450 monooxygenase (P450s). Forty-six genes (1.4%) were coregulated between Rd-5 and Rd-40, including scavenger receptors and uncharacterized proteins (Fig. S2).

### Validation of RNA-seq gene expression levels

**Fig. 5:**
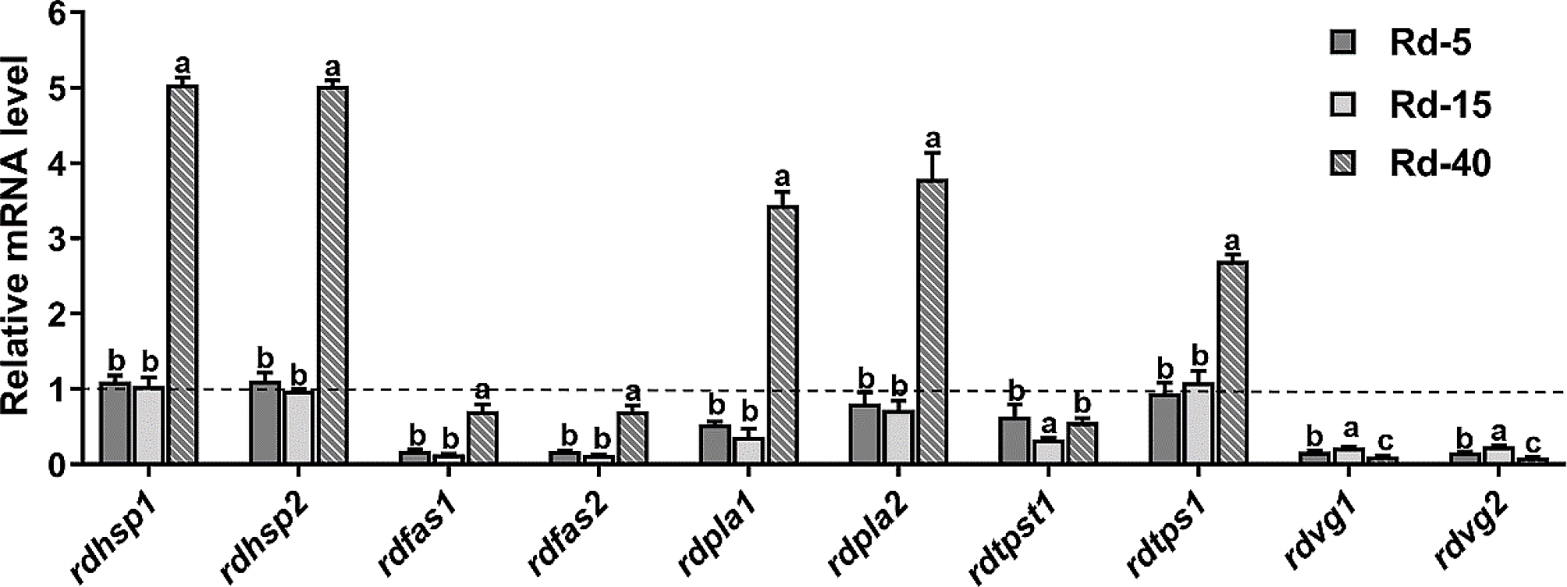
Expression of representative selected genes was determined in various samples of *Rhyzopertha dominica*. Different letters (a to c) above the bars indicate a significant difference (*P* < 0.05) by the Tukey test. The expression level of each gene in control Rd-ck was mathematically designated as 1 in each reaction

To validate the RNA-Seq results, a subset of DEGs was randomly selected for RT-qPCR. The correlation coefficient R^2^ was 0.8782, as determined by linear regressions of fold change differences, which indicated that the RT-qPCR and RNA-seq results were consistent (Fig. S3). The RNA-Seq results suggested that certain genes, including those coding for HSPs, FAS, phospholipases, trehalose transporter (TPST), trehalose-6-phosphate synthase (TPS), and Vg, were differentially expressed under temperature stress. Hence, we evaluated the expression levels of two HSPs (*rdhsp1*, *rdhsp2*), two fatty acid synthesis (*rdfas1*, *rdfas2*), two phospholipases (*rdpla1*, *rdpla2*), one trehalose transporter (*rdtpst1*), one trehalose-6-phosphate synthase (*rdtps1*), and two vitellogenin (*rdvg1*, *rdvg2*) using RT-qPCR analysis with *rps3* (a ribosomal protein S3 gene) as a reference gene. The expression of the genes encoding HSPs, phospholipases, and trehalose-6-phosphate synthase increased significantly under heat stress. Specifically, FAS and Vg were down-regulated under both heat and cold stress (Fig. 5). These results confirmed the different regulatory mechanisms that the lesser grain borers undergo when exposed to low and high temperature conditions.

### **Silencing*rdtps1*significantly reduced the survival rate of*Rhyzopertha dominica*at high temperature**

**Fig. 6:**
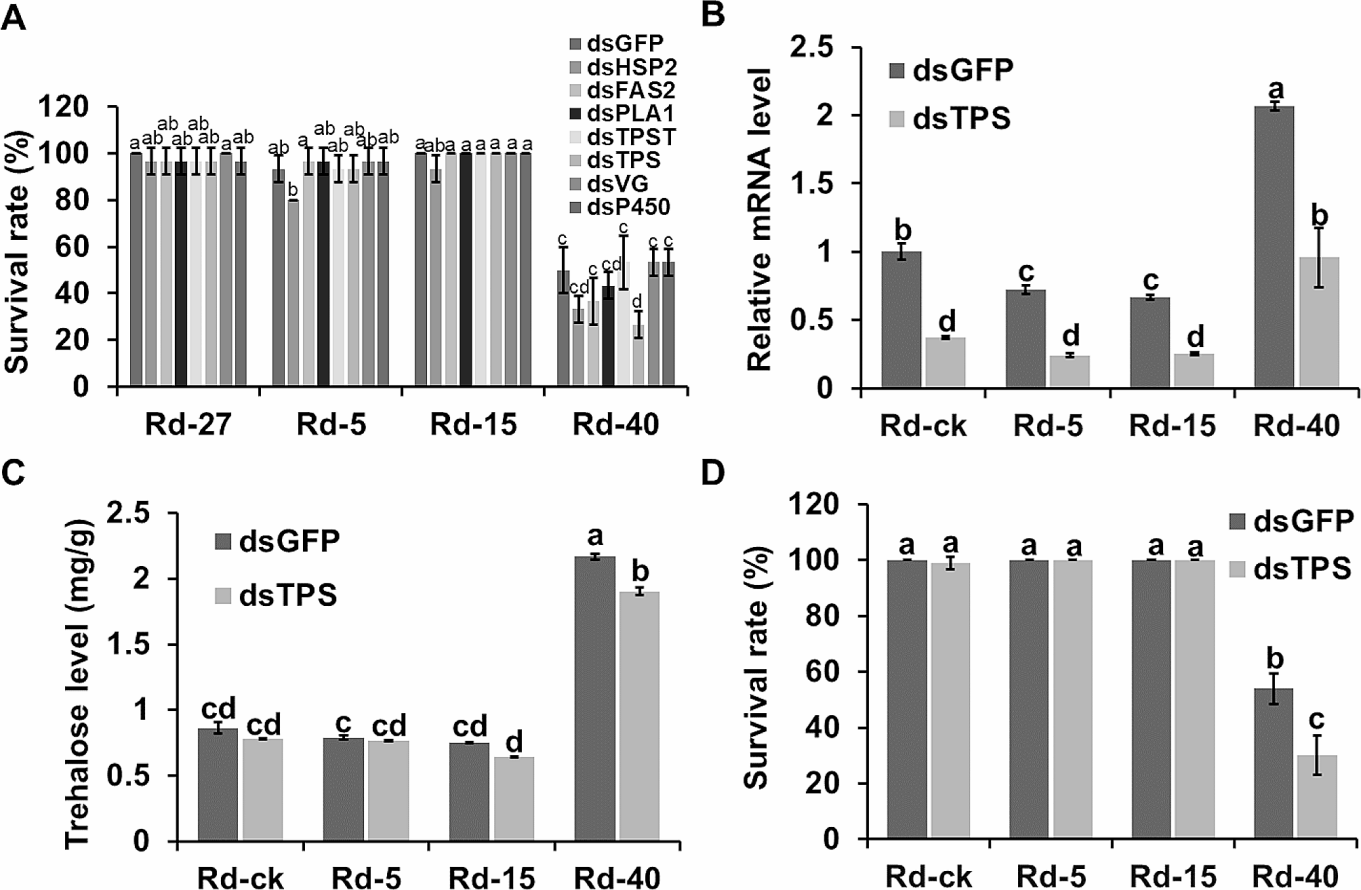
The *rdtps-1* Silencing impact on the survival rate of *Rhyzopertha dominica* at high temperature. **(A)** The effect of silencing 7 candidate genes on the survival rates after 7 days of temperature treatments. **(B)** Effect of RNAi-mediated silencing on *rdtps1* expression levels determined in various samples by using RT-qPCR. **(C)** The trehalose content of each sample was tested by anthrone colorimetric method. The insects were fed on dsGFP or dsTPS for two days and then raised at different temperatures for two days. **(D)** Survival rates after 7 days of temperature treatments were recorded and calculated. Different letters (a to d) above the bars indicate a significant difference (*P* < 0.05) by the Turky test

To further investigate the function of the DEGs, RNAi assay was performed on seven candidate genes (*rdhsp1*, *rdfas1*, *rdpla1*, *rdtpst1*, *rdtps1*, *rdvg1*, and a P450 encoding gene) at different temperatures (Fig. 6A). The results indicated that interference with the expression of *rdtps1* had a significant impact on pest mortality under high temperature conditions. Then, we determined the silencing efficiency of RNAi by examining the relative expression level of *rdtps1* via RT-qPCR. The results showed that compared to the negative control (dsGFP), the dsTPS treatment effectively reduced the *rdtps1* expression even with the elevated *rdtps1* expression at 40 °C (Fig. 6B). Since *rdtps1* is a predicted trehalose synthetase, we measured the trehalose level in each sample. Among the dsGFP controls, the trehalose level in the Rd-40 increased significantly compared with Rd-ck, while that of Rd-5 and Rd-15 did not change significantly. When the insects were reared at 40 °C with dsTPS, the trehalose level decreased significantly (Fig. 6C). These results showed that Rdtps1 is an important trehalose synthase that responds to high temperature stress. (Fig. 6D). However, we did not observe a reduced survival rate in the other dsTPS treated groups at normal or low temperature (Fig. 6D). These results demonstrated that *rdtps1* responded to heat stress and expression level of this gene was crucial for pest survival at high temperature.

## Discussion

*Rhyzopertha dominica* is one of the most temperature-adaptive insects among stored product pests. Transcriptome analysis, as an important means of studying the molecular mechanisms by which pests resist temperature stress, has also been applied multiple times in stored grain pests, such as in the studies of *Tribolium castaneum* [13] and *C. chinensis* [15]. In this study, our transcriptome analysis revealed that *R. dominica* regulated numerous genes to respond to temperature stress. The average length of the transcripts was 2,806 bp, which indicated that the data were good enough to represent full-length transcripts [37]. Using the RT-qPCR analysis and RNAi assay, we characterized thatthe trehalose-phosphate synthase encoding gene *rdtps1* positively regulates the high temperature resistance of *R. dominica*. Trehalose is an important carbohydrate in insect hemolymph, and trehalose-phosphate synthase (TPS) is responsible for the synthesis of trehalose [38]. Thermal acclimation led to an increase in trehalose level, suggesting the involvement of trehalose in temperature response regulation in *R. dominica*. Furthermore, *rdtps1* was highly expressed at elevated temperature, indicating its role in trehalose synthesis. When the gene expression of *rdtps1* was repressed by RNAi, both the trehalose content and survival rate decreased significantly. This highlights the importance of *rdtps1* as an important regulatory factor for *R. dominica* in response to high temperature stress. Additionally, under moderate or low temperature stress conditions, interfering with the expression of *rdtps1* did not alter trehalose levels and the survival rate of pests, indicating that *R. dominica* have different regulatory pathways in response to high and low temperature stress.

In the RNAi assay, we silenced seven DEGs, including *rdhsp1*, *rdfas1*, *rdpla1*, *rdtpst1*, *rdtps1*, *rdvg1*, and a P450 encoding gene (*rdP450*). However, silencing only *rdtps1* resulted in increased insect mortality. These findings suggested that *rdtps1* is more important for the pest heat resistance compared to the other six genes. Alternatively, it is also possible that the function of these genes can be compensated by other metabolic pathways. Further validation is required to elucidate the functions of the other genes. When we silenced *rdtps1*, we observed a 50% increase in mortality, although trehalose level of the insects only reduced 10%. This observation suggested that the heat resistance of *R. dominica* requires a high level of trehalose. However, we cannot exclude the possibility that reduced *rdtpst1* gene expression has a systemic impact on the metabolism of the pest under high temperature conditions. Further research is needed to determine the effect of reduced gene expression. In addition, transcriptome sequencing revealed that approximately 30 other genes were putative TPS genes. Although these predicted TPS genes did not exhibit upregulated expression under high-temperature conditions, they may compensate for the significant reduction in *rdtps1* expression and maintenance of the trehalose levels in the pests. Future studies of these genes are also necessary.

At 40 °C, the average weight of the pests significantly decreased 31% after being fed for 7 days. This may be due to the intensified metabolism caused by high temperature, which results in the consumption of fat by pests. For instance, the gene expression level of the putative helicase (B3-1_1-10k_transcript/13,726) was upregulated at 40 °C in the transcriptome data. The B3-1_1-10k_transcript/13,726, which catalyzes the unwinding of double-stranded nucleic acids using energy from ATP, is in the spliceosome pathway (ko03040). Aldehyde dehydrogenase (B3-1_1-10k_transcript/14,337) participants in fatty acid degradation and produces a large amount of ATP (ko00071). Moreover, the expression of fatty acid synthase (ko00061) and acyl-CoA delta (11) desaturase (ko01212), which are responsible for synthesizing fat, was down-regulated, leading to the inhibition of fat synthesis. Consequently, the average weight of pests decreased significantly under high temperature conditions. Although low temperature also resulted in the down-regulation of fatty acid synthase expression, it caused pests to become unconscious and suppressed metabolism, leading to a reduction in fat consumption.

Previous studies have shown that several other genes are involved in temperature regulation, although little is known about this topic in *R. dominica*. Numerous studies have indicated that the expression of HSPs significantly changes under temperature stress to ensure the configuration stability of proteins and to repair DNA damage [39–46]. As such, HSPs are also vital for the temperature response of insects. In our study, the expression of HSPs, including HSP70, HSP90, and small HSPs, was upregulated in Rd-40 (40 °C), which indicated that the HSPs of *R. dominica* play an important role in the response to high temperature. In addition to HSPs, heat stress also led to high expression of phospholipases encoding genes *rdpla1* and *rdpla2* in *R. dominica*, and phospholipases are essential enzymes for cell membrane stability [47]. In *Apolygus lucorum*, phospholipase regulates trehalose in 20-hydroxyecdysone-induced fecundity [48], and our transcriptome analysis and RT-qPCR results showed that the coding genes of vitellogenin (*rdvg1, rdvg2*) were also significantly downregulated under temperature stress conditions. Vitellogenin, the precursor of vitellin, is an indicator of oviposition ability and reproduction in most oviparous species including insects [49–52]. Vitellogenin is stored in eggs to provide a source of nutrients for the early development of embryos and larvae. We hypothesized that reducing the expression of vitellogenin by pests is also a strategy for helping the lesser grain borers cope with temperature stress, reducing energy consumption while preventing offspring from facing adverse environmental conditions. These results indicate that the lesser grain borers employed multiple strategies to respond to temperature stress.

## Conclusions

We thoroughly explored the regulatory genes involved in the response of lesser grain borers to temperature changes through transcriptome sequencing. The results showed that the gene expression profiles of insects reared at high temperature changed dramatically compared to those reared at ambient temperature. The metabolic pathways co-regulated by temperature stresses (5 °C, 15 and 40 °C) were mainly enriched in “fatty acid metabolism”, “fatty acid biosynthesis”, “starch and sucrose metabolism”, “neuroactive ligand-receptor interaction” and “protein digestion and absorption”. The DEGs under cold stress ((5 °C, 15 °C) mainly encoded fatty acid synthase and protein kinases. The DEGs under heat stress (40 °C) were associated primarily with heat shock proteins (HSPs), ATP-binding cassettes, and phospholipases. Thirty-four genes, which are mainly encoding genes of FASs and Vgs, were co-regulated under both cold and heat stress. RNAi assay of seven candidate genes (*rdhsp1*, *rdfas1*, *rdpla1*, *rdtpst1*, *rdtps1*, *rdvg1*, and *rdP450*) revealed that the coding gene of trehalose synthase (*rdtps1*) played a crucial role in regulating the heat resistance of *R. dominica*. This study provides insights into the molecular biology of *R. dominica* and contributes to the identification of a novel target gene for heat resistant pest control.

## Methods

### Insects and samples

*Rhyzopertha dominica* were collected from the granary of Guangzhou (23.13 N 117.19 E) in southern China. The insects were maintained in the laboratory and reared on wheat at 27 ± 0.1 °C, 65 ± 0.5% relative humidity (RH) in an incubator (Memmert, Germany) for at least three years.

We selected two-week-old adults for the assays. All the individuals were maintained at the same relative humidity (65% RH). We set four temperature conditions, including 5 °C, 15 °C, 27 °C, and 40 °C. *R. dominica* kept at 27 °C was used as the control group (Rd-ck), whereas *R. dominica* kept at 5 °C, 15 °C, and 40 °C were the treatment groups (Rd-5, Rd-15, and Rd-40).

### Laboratory bioassays

For the mortality assay, 30 individuals for each treatment of Rd-ck, Rd-5, Rd-15, and Rd-40 were reared for 7 days. The number of dead insects was recorded each day and used to calculate the mortality rate of *R. dominica*. The experiments were repeated five independent times.

To measure the effects of different temperatures on the average weight over a period of 7 days, we weighed 30 individuals of *R. dominica* for each temperature condition. Each individual was fed a clean grain of wheat in a separate well on a 24-well plate for 7 days. The individuals’ weight was measured by an analytical balance (ME204E, Mettler Toledo, China) to assess weight change. The experiments were repeated three independent times.

### Sample preparation

For ISO-Seq, 10 adults of the Rd-ck were selected, and then immediately frozen in liquid nitrogen. For RNA-Seq, 3 samples of each treatment group, Rd-ck, Rd-5, Rd-15, and Rd-40, were processed, and each sample included 10 adults. The insects were reared for 2 days and then frozen in liquid nitrogen. Total RNA was extracted using an RNeasy Plus Mini Kit (Qiagen, Germany), following the manufacturer’s instructions. RNA degradation and contamination were assessed on agarose gels. To determine the concentration and purity of the total RNA, we added 1 µL of RNA to a NanoDrop 2000 Spectrophotometer (Thermo Fisher, USA). RNA integrity was evaluated using an Agilent Technologies 2100 Bioanalyzer (Agilent, USA).

### Library construction, sequencing, and data quality control for PacBio full-length ISO-Seq

Third-generation Isoform-sequencing sequencing via PacBio can be used to obtain high-quality single full-length transcriptome sequences without assembly. The average read length is 8–15 kb, making it easy to cross the full transcript. It can accurately identify isomers and perform precise analysis of variable splicing, fusion genes, homologous genes, superfamily genes, and allele expression. We used magnetic beads with an oligo(dT) primer to enrich the mRNA of the samples. Then, full-length cDNA was synthesized using a SMARTer™ PCR cDNA Synthesis Kit (Clontech, USA). The cDNA was amplified and enriched by PCR. To screen full-length cDNA fragments, we used BluePippin (Sage Science, USA) and constructed libraries based on the length of the insert fragments. Sequencing was performed on a PacBio Sequel (Pacific Biosciences, USA), and the raw data output was 20 Gb.

To obtain full-length transcripts, the raw data were filtered to remove linker sequences, fragments < 300 bp, and sequences with an accuracy < 0.9. Then, the raw reads with the number of full passes ≥ 1 and sequence accuracy > 0.9 were screened to obtain the raw reads. The transcripts were subsequently obtained through classification, clustering, and correction processes. Since there may be many repeated transcripts, we clustered the transcripts using an isoform-level clustering (ICE) algorithm. Similar sequences were clustered into a cluster, and each cluster contained a consistent transcript. Combined with non-full-length sequences, the polish program was used to correct the raw reads in each cluster. We obtained high-quality transcripts (HQ, high-quality isoforms) and low-quality transcripts (LQ, low-quality) transcripts with an accuracy > 99%. The data from raw RNA-Seq (Illumina NGS) were used to correct LQ full-length transcript isoforms using proofreading software. For the full-length transcriptome, CD-HIT software was used to capture de-redundant sequences in the high-quality full-length transcriptome (parameters: C = 0.99, T = 6, G = 1, U = 10, S = 0.999, D = 40, *P* = 1) [53], resulting in a non-redundant high-quality transcriptome, which is the full-length transcripts. The transcriptome raw data were deposited to the NCBI SRA database (accession number: PRJNA733562).

### Annotation of transcripts

Functional annotation was performed via comparison with public databases. The NCBI nucleotide sequences (NT) is the nucleotide database of NCBI (https://www.ncbi.nlm.nih.gov/), which includes the nucleotide sequences in GenBank, the European Molecular Biology Laboratory (EMBL), and the DNA Data Bank of Japan (DDBJ). The NCBI non-redundant protein sequences (NR) is the protein sequence database of NCBI. Swiss-Prot (http://www.ebi.ac.uk/uniprot/) is a manually annotated and reviewed protein sequence database of protein sequences sorted and studied by biologists. Gene Ontology (GO) (http://geneontology.org/) is an internationally standardized gene function classification system that provides a set of dynamically updated controlled terms to comprehensively describe the attributes of genes and gene products in organisms. The Kyoto Encyclopedia of Genes and Genomes (KEGG) (http://www.genome.jp/kegg/) is a database that systematically analyses metabolic pathways and functions of gene products and compounds in cells. Clusters of orthologous groups for Eukaryote (KOG) (https://ftp.ncbi.nih.gov/pub/COG/KOG/) possesses clusters of orthologous groups of proteins in eukaryotes according to their phylogenetic classification.

### Library construction, sequencing, and data quality control for RNA-Seq

For NGS, libraries were generated using the VAHTS Stranded mRNA-seq Library Prep Kit for Illumina V2 (Vazyme Biotech, China). The libraries were subsequently sequenced on an Illumina NovaSeq platform to generate 150-bp paired-end reads. The raw data of RNA-Seq were first processed through primary quality control. Clean reads were obtained by removing read pairs. The data filtering procedure was as follows: (1) When *N* > 3 in any end sequencing read, this pair of paired reads was removed; (2) when the proportion of bases with quality value < 5 was > = 20% at any end, this pair of paired reads was removed; (3) when the adapter sequence was removed, it was necessary to match at least 8 bp. The RNA-Seq transcriptome raw reads were deposited to the NCBI SRA database (accession number: PRJNA734384).

### Differentially expressed genes analysis

Using the full-length transcript (ISO-Seq data) as a reference sequence, we mapped the clean reads (RNA-Seq data) of each sample to the reference sequence by RSEM [54]. In this process, the mismatch parameter of Bowtie2 [55] is 0. RSEM was used to analyzlyse the comparison results obtained with Bowtie software, and the read counts of each gene were further obtained for each sample. Next, we converted the read count into FPKM (the expected number of fragments per kilobase of transcript sequence per million base pairs sequenced) to analyze the expression level of each gene. To analyze the DEGs in each sample, we used the package edgeR [56] of RStudio [57], and differentially expressed P and FDR values were calculated. FDR is the corrected P value. The smaller the FDR value is the more significant the difference in gene expression. The screening conditions were as follows: FDR < 0.05 and log_2_|fold-change| > 1.

### Reverse transcription quantitative real-time PCR (RT-qPCR)

HiScript® III RT SuperMix for qPCR (Vazyme Biotech, China) was used to synthesize cDNA. Reverse transcription quantitative real-time PCR (RT-qPCR) was performed using 2 ng of cDNA and AceQ® Universal SYBR® qPCR Master Mix (Vazyme Biotech, China) on a Bio-Rad CFX real-time PCR system. Each 20 µL reaction consisted of 10 µL of 2×ChamQ Universal SYBR qPCR Master Mix, 5 µL of 1:100 diluted cDNA, 0.4 µL for forward and reverse primers (10 µM), and 4.2 µL of RNase-free ddH_2_O. The RT-qPCR reaction cycle consisted of a denaturation step at 95 °C for 30 s, amplified with 40 cycles of 95 °C for 10 s, 60 °C for 30 s, and ending with a melting-curve step. 10 DEGs genes were selected and the relative levels of gene expression were determined using the threshold cycle 2^−ΔΔCT^ method, with the ribosomal protein S3 gene (*rps3*) serving as the reference gene [58]. In this study, RT-qPCR primers were designed with Primer 5 (Table 2). Three technical replicates were used each time.

**Table 2:** Primers used in this study

Primer Name	Sequences (5’→3 ‘)	Length	Gene	Gene ID	Purpose	
Rdhsp1-F	GACGGAATCGACTTCTACACC	129	*rdhsp1*	B3-1_1-10k_transcript/15,170	RT-qPCR	
Rdhsp1-R	GATTGACCCTTTGTCCATTTT					
Rdhsp2-F	CAACAACCTCTTGGGTACTTTCG	120	*rdhsp2*	B3-1_1-10k_transcript/15,497	RT-qPCR	
Rdhsp2-R	TCCTTCGCAGATACGTTCAGG					
Rdfa1-F	GTACTCATTCACGCTGGTAGTGG	104	*rdfa1*	B3-1_1-10k_transcript/10,065	RT-qPCR	
Rdfa1-R	CTCTTTGCTTGACTGCCGACT					
Rdfa2-F	CGGTACTCATTCACGCTGGTA	106	*rdfa2*	B3-1_1-10k_transcript/1385	RT-qPCR	
Rdfa2-R	CTCTTTGCTTGACTGCCGACT					
Rdpla1-F	CTTCTGCTGGTGCCTATT	97	*rdpla1*	B3-1_1-10k_transcript/9998	RT-qPCR	
Rdpla1-R	CGAAAGTCGTATGGTGCT					
Rdpla2-F	ATACCAGGATTCGTTTGG	96	*rdpla2*	B3-1_1-10k_transcript/22,992	RT-qPCR	
Rdpla2-R	TTCTTGATGGATAGGGACT					
Rdtpst1-F	GTGGCACAAGATAACAACAGA	145	*rdtpst1*	B3-1_1-10k_transcript/20,862	RT-qPCR	
Rdtpst1-R	GAGAAGCGGCAGGTAGAA					
Rdtps1-F	TAAAGGTGCCCTGCTGAC	100	*rdtps1*	B3-1_1-10k_transcript/7670	RT-qPCR	
Rdtps1-R	GAACCCTGCTTCTTCAATC					
Rdvg1-F	CCCATAATGAACAGTCCACAA	140	*rdvg1*	B3-1_1-10k_transcript/14,375	RT-qPCR	
Rdvg1-R	TGATACAATCTTCCGTAATCCA					
Rdvg2-F	TCGTCAGCGACAGCCAGGTC	102	*rdvg-2*	B3-1_1-10k_transcript/26,394	RT-qPCR	
Rdvg2-R	TCTTGTGCGGAGCGCAGTTC					
Rdrps3-F	TGGGACCCTAATGGCAAGA	137	*rdrps3*	B3-1_1-10k_transcript/26,326	RT-qPCR	
Rdrps3-R	GCAACAGGTGGTATGTCTGATTT					
dsTPS-F	GGATCCTAATACGACTCACTATAGGTCTACAGGGATGCTGCAGTG	486	*rdtps1*	B3-1_1-10k_transcript/7670	RNAi	
dsTPS-R	GGATCCTAATACGACTCACTATAGGCCAAATCAGGATGAGGTGCT					
dsGFP-F	GGATCCTAATACGACTCACTATAGGGATTAAGTTCAGCGTGTCCG	428	*gfp*	(Qiu et al., 2015)	RNAi	
dsGFP-R	GGATCCTAATACGACTCACTATAGGCTAGTGATTCACCTTGATGC					
dsHSP2-F	GGATCCTAATACGACTCACTATAGGGGGCACGATTCGAAGAATTA	445	*rdhsp2*	B3-1_1-10k_transcript/15,497	RNAi	
dsHSP2-R	GGATCCTAATACGACTCACTATAGGCTCTCACCCTCGAAGACCTG					
dsFAS2-F	GGATCCTAATACGACTCACTATAGGTATTGGAAATTCGCGTGACA	465	*rdfa2*	B3-1_1-10k_transcript/1385	RNAi	
dsFAS2-R	GGATCCTAATACGACTCACTATAGGGCAGTGACCGTTTTTGGATT					
dsPLA1-F	GGATCCTAATACGACTCACTATAGGTACCTGGTGATGGTGGTTCA	493	*rdpla1*	B3-1_1-10k_transcript/9998	RNAi	
dsPLA1-R	GGATCCTAATACGACTCACTATAGGTACAAAGACATTGGGCCTCC					
dsTPST-R	GGATCCTAATACGACTCACTATAGGGCCGCTTCTCTTGTTTTGAC	441	*rdtpst1*	B3-1_1-10k_transcript/20,862	RNAi	
dsTPST-F	GGATCCTAATACGACTCACTATAGGCATCCCGATACCAGAGAGGA					
dsVG-F	GGATCCTAATACGACTCACTATAGGATATCTACCCACTGCCACGC	425	*rdvg1*	B3-1_1-10k_transcript/14,375	RNAi	
dsVG-R	GGATCCTAATACGACTCACTATAGGCTTCTGCACGGTGAAACAGA					
dsP450-F	GGATCCTAATACGACTCACTATAGGTTGATCCTTTCATGGGTGGT	411	*rdP450*	B3-1_1-10k_transcript/18,550	RNAi	
dsP450-R	GGATCCTAATACGACTCACTATAGGTGCAATCCTTTGGGAAGAAC					

### RNAi assay

A 486 bp fragment of the *rdtps1* gene was selected for RNAi assay. Double-stranded RNA (dsRNA) was synthesized using the T7 RiboMAX™ Express RNAi System (P1700, Promega, USA). The primers dsTPS-F and dsTPS-R were used to synthesize the dsTPS. To eliminate any potential effects from experimental procedures or nanocarriers, a 428bp fragment of the enhanced green fluorescent protein gene (*gfp*) was separately constructed [59], and dsGFP-F and dsGFP-R were used to synthesize the negative control dsGFP (Table 2). The dsRNA was mixed with the nanocarrier at the recommended mass ratio of 1:1 in RNase-free water. Then, the dsRNA solution was added to wheat flour to make feed for the adults of *R. dominica.* Adults were fed on the feed with dsRNA at 27 °C for 2 days and then were incubated at 5, 15, 27 or 40 °C for 2 days. The silence efficiency was examined by RT-q-PCR of the primers was evaluated via RT-q-PCR using *rps3* as the reference gene [58]. Three technical replicates were used each time. The trehalose content of each treatment was determined using 30 individuals by Trehalose Content Assay Kit (BC0355, Solarbio, China). Three technical replicates were used each time. After 7 days of treatment at different temperatures, 30 individuals were observed. and the survival rate was calculated. Five technical replicates were used each time.

### Statistical analysis

The means and standard deviations of the experimental results were calculated using Excel. Tukey test was performed using the Statistical Package for the Social Sciences (SPSS) version 27.0 (IBM SPSS Statistics, Armonk, NY, USA).

### Electronic supplementary material

Below is the link to the electronic supplementary material.

Supplementary Material 1

## Data Availability

The sequencing reads are available from the Sequence Read Archive (SRA) under the BioProject accession numbers PRJNA733562 and PRJNA734384. For additional data and custom scripts, please contact the corresponding author.

## Abbreviations

DEGs: Differentially expressed genes
TPS: Trehalose-6-phosphate synthase
TPP: Trehalose-6-phosphate phosphatase
HSPs: Heat shock proteins
FAS: Fatty acid synthase Vg:vitellogenin
P450s: Cytochrome P450 monooxygenase
