# Supplementary material for: Trehalose 6-phosphate synthase gene rdtps1 contributes to thermal acclimation in Rhyzopertha dominica
Source: BMC Genomics. 2024 Feb 13;25:172. doi: 10.1186/s12864-024-10028-4 (PMC10863172; doi:10.1186/s12864-024-10028-4)
Supplement: Supplementary file 1 — Supplementary Material 1 [file 12864_2024_10028_MOESM1_ESM.docx]

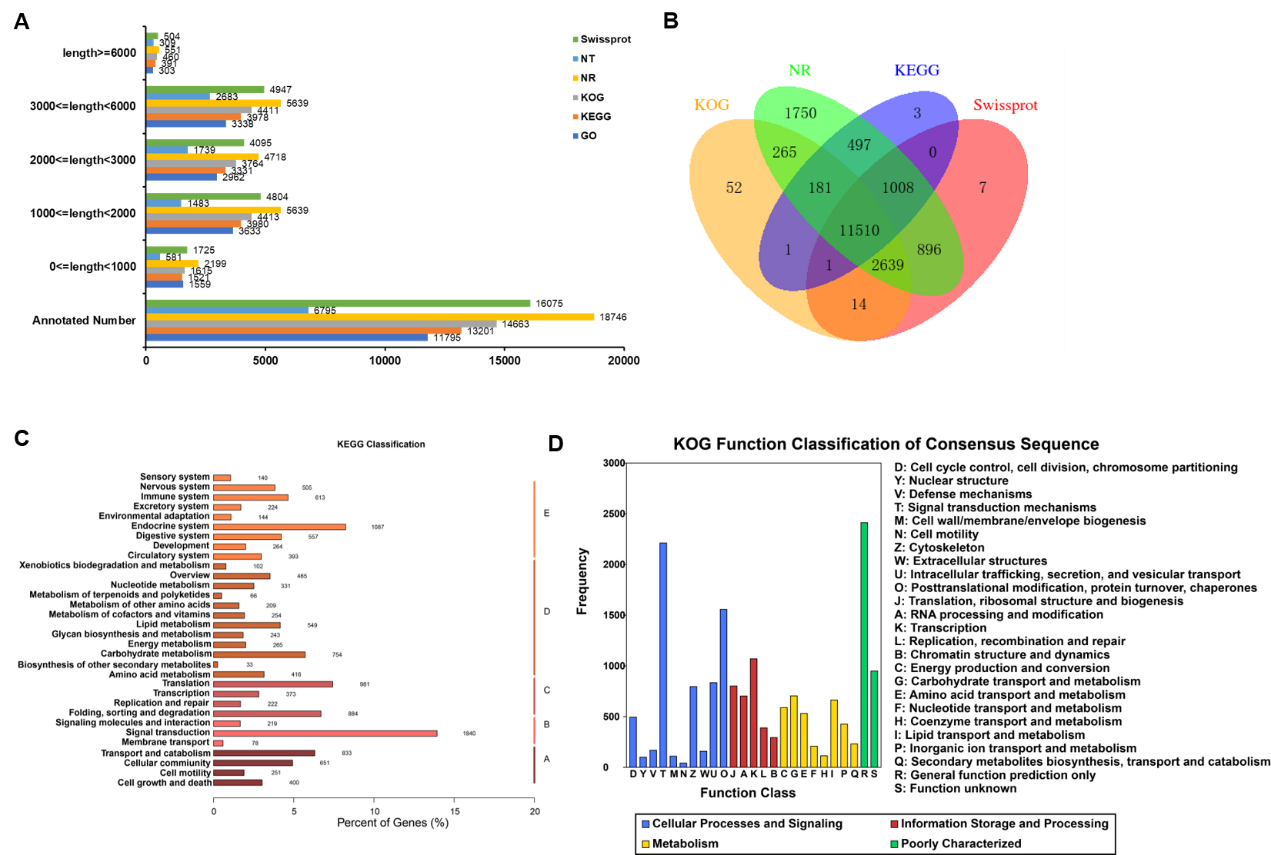


**Fig. S1.** Transcript functional annotations of *Rhyzopertha dominica*. (A) The annotation numbers of full-length transcripts of different lengths analyzed by six public databases including Swiss-Prot (A manually annotated and reviewed protein sequence database), NT (NCBI nucleotide sequences), NR (non-redundant proteins), KOG (Clusters of orthologous groups for Eukaryote), KEGG (Kyoto Encyclopedia of Genes and Genomes), and GO (Gene Ontology). (B) Venn diagram of all isoforms from the reference transcriptome hits of KOG, NR, KEGG and Swiss-Prot. (C) KEGG classification of all identified isoforms from the reference transcriptome. A, B, C, D, and E indicated cellular processes, environmental information processing, genetic information processing, metabolism, and organismal systems. (D) KOG classification using the reference transcriptome.


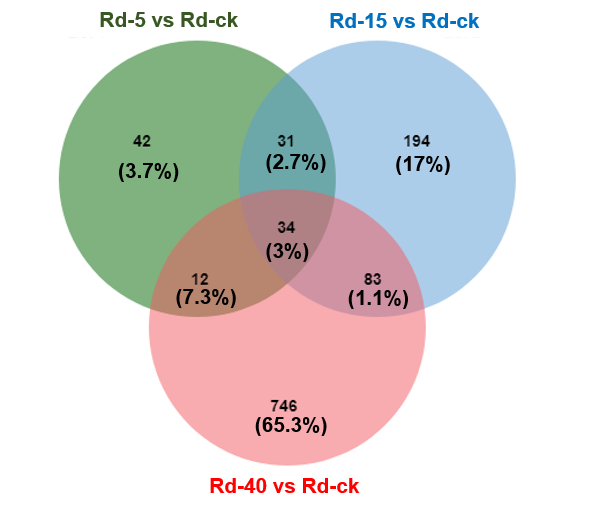


**Fig. S2.** Venn diagram of Differentially expressed genes (DEGs) of *Rhyzopertha dominica* exposed to different temperatures.


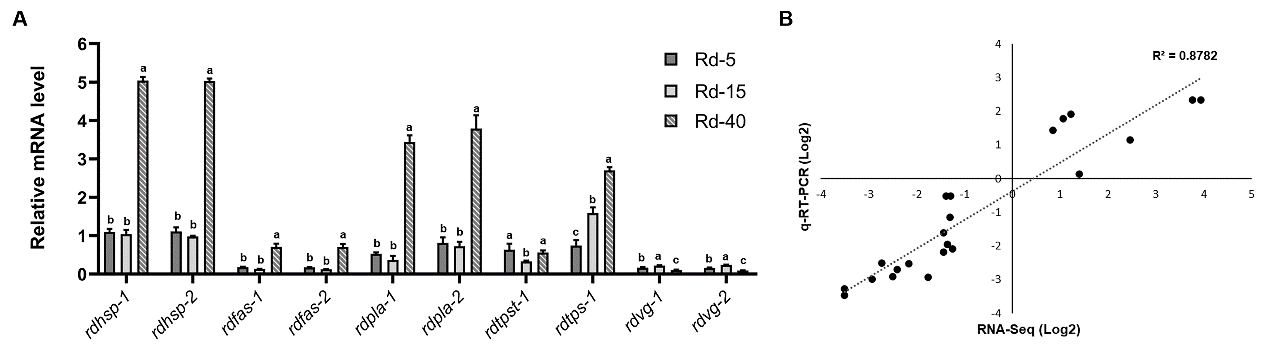


**Fig. S3.** Validation of RNA-seq data by RT-qPCR assays. log_2_(FC) of gene expression detected by RNA-seq were plotted against the data of RT-qPCR. The reference line indicates the linear relationship between the results of RNA-seq and RT-qPCR. R^2^ is coefficient of determination.
